# Supplementary material for: Variable expression of eighteen common housekeeping genes in human non-cancerous kidney biopsies
Source: PLoS One. 2021 Dec 9;16(12):e0259373. doi: 10.1371/journal.pone.0259373 (PMC8659319; doi:10.1371/journal.pone.0259373)
Supplement: Attachment — Submitted filename: Response to Reviewers.doc [file pone.0259373.s003.doc]

Bergen, October 2021

Stephen D. Ginsberg, Ph.D.
Section Editor
PLOS ONE

Dear editor and reviewers

Thank you for taking the time to review our work a second time. We have taken your comments to heart and improved the manuscript accordingly. Below you will find a point-by-point review of the changes to the paper in response to each comment.

**Reviewer #2:**

**1. Reviewer #2:** I think the paper would benefit from an explicit example of
what the effect would be on variation in relative gene expression using a a
less stable vs more stable reference gene.

This could even be a theoretical discussion just looking at effect of
variation in the denominator ie reference gene.
I think this would make the paper eve more impactful and drive the points
home about housekeeper gene stability

**Response to 1. Reviewer #2:** We thank the reviewer for taking the time to review the manuscript again. We have added a section on the suggested topic to the discussion. See lines 401 – 411.

To conclude this letter, we would like to thank again all reviewers for their valuable comments and taking the time to review the manuscript again. We hope to have answered all issues satisfactory.

Sincerely,

Philipp Strauss

University of Bergen

Department of Clinical Medicine

Haukeland University Hospital

Jonas Lies Vei 65,

Laboratory Building, 7th floor

5021 Bergen, Norway

Mobile phone: 0047 93686433

E-mail: [Philipp.Strauss@uib.no](mailto:Philipp.Strauss@uib.no)
